# Low Hepcidin Levels in Severely Anemic Malawian Children with High Incidence of Infectious Diseases and Bone Marrow Iron Deficiency

**DOI:** 10.1371/journal.pone.0078964

**Published:** 2013-12-05

**Authors:** Femkje A. M. Jonker, Job C. J. Calis, Kamija Phiri, Rob J. Kraaijenhagen, Bernard J. Brabin, Brian Faragher, Erwin T. Wiegerinck, Harold Tjalsma, Dorine W. Swinkels, Michael Boele van Hensbroek

**Affiliations:** 1 Global Child Health Group, Emma Children's Hospital, Academic Medical Centre, Amsterdam, The Netherlands; 2 Community Health Department, College of Medicine, Blantyre, Malawi; 3 Meander Medical Centre, Laboratory for Clinical Chemistry and Haematology, Amersfoort, The Netherlands; 4 Liverpool School of Tropical Medicine, University of Liverpool, United Kingdom; 5 Department of Biostatistics Liverpool School of Tropical Medicine, University of Liverpool, United Kingdom; 6 Laboratory Medicine, Laboratory of Genetic, Endocrine and Metabolic Diseases, Radboud University Nijmegen Medical Centre, Nijmegen, The Netherlands; 7 Hepcidinanalysis.com, 6525 GA Nijmegen, The Netherlands; University of Massachusetts Medical School, United States of America

## Abstract

**Introduction:**

A reliable diagnostic biomarker of iron status is required for severely anemic children living in malarious areas because presumptive treatment with iron may increase their infection risk if they are not iron deficient. Current biomarkers are limited because they are altered by host inflammation. In this study hepcidin concentrations were assessed in severely anemic children living in a highly malarious area of Malawi and evaluated against bone marrow iron in order to determine the usefulness of hepcidin as a point of care test.

**Methods:**

207 severely anemic children were assessed for levels of hepcidin, ferritin, serum transferrin receptor, erythropoietin, hematological indices, C-reactive protein, interleukin-6, malaria parasites and HIV infection. Deficiency of bone marrow iron stores was graded and erythroblast iron incorporation estimated. Interaction of covariates was assessed by structural-equation-modeling.

**Results and Conclusion:**

Hepcidin was a poor predictor of bone marrow iron deficiency (sensitivity 66.7%; specificity 48.5%), and of iron incorporation (sensitivity 54.2%; specificity 61.8%), and therefore would have limitations as a point of care test in this category of children. As upregulation of hepcidin by inflammation and iron status was blunted by erythropoietin in this population, enhanced iron absorption through the low hepcidin values may increase infection risk. Current recommendations to treat all severely anemic children living in malarious areas with iron should therefore be reconsidered.

## Introduction

Iron deficiency is a nutritional disorder highly prevalent among pre-school children living in developing countries [1] and as a major contributor to child morbidity [2], [3], iron supplementation programs have been prioritized [4]. In view of concerns that iron supplementation may increase malaria related morbidity and mortality, especially in iron replete children, [5], [6], the World Health Organization (WHO) recommended diagnosis of iron deficiency prior to supplementation [6].This recommendation is problematic in malarious areas as a reliable bio-marker to identify iron deficiency is not available due to the effects of inflammation on biomarker concentrations. The WHO also advises iron treatment of children with signs of severe anemia [6]. Yet a large proportion of severely anemic children living in highly malarious areas may be iron replete [7] and iron presumptive treatment may enhance their infection risk. A peripheral blood iron biomarker for reliable iron status assessment is required to ensure safety of preventive and curative iron interventions.

The iron regulator hepcidin [8]–[10] plays a central role in the interaction between iron deficiency, anemia and inflammation [11]–[13]. In iron loaded conditions and during inflammation/infection increased hepcidin levels cause a decrease in intestinal iron absorption, and iron release from storage through degradation of the cellular iron exporter ferroportin. [11], [12], [14], [15]. Conversely, iron deficiency, hypoxia and enhanced erythropoiesis decrease hepcidin levels, [12], [14], [16] leading to an increase in iron levels. In view of its central role in iron homeostasis serum hepcidin could be a useful marker to guide iron supplementation interventions, even in areas with high infection exposure [17]–[19], although it has yet to be evaluated for this purpose [20]. In this study we evaluated serum hepcidin as a predictor of deficient bone marrow iron stores, and erythroblast iron incorporation in severely anemic Malawian children. Impact on serum hepcidin of iron levels, inflammation, hypoxia and erythropoiesis was also assessed.

## Patients and Methods

### Study design and population

This study formed part of a large research program conducted between 2002–2004 investigating the etiology, pathogenesis and outcome of severe anemia in Malawian children. A detailed description of the study methodology is available [7], [21]. In brief, 381 children (6–60 months old) presenting with severe anemia (hemoglobin<5.0 g per deciliter) were enrolled as ‘cases’ if they had not been transfused in the previous 4 weeks. For each case, two controls were enrolled, a community-control living within 100–1000 meters of the case, and a hospital-control, presenting at the same hospital or outpatient facility as the case. Controls were eligible for recruitment if they were aged between 6–60 months and if their hemoglobin level was 5.0 gram per deciliter or more. At recruitment a venous blood sample was obtained and, (in cases) if the clinical condition allowed, a bone marrow aspiration was performed.

For the hepcidin study, all cases with available bone marrow aspirates were included. In order to compare hepcidin levels between severely anemic and non-severely anemic children, hepcidin was also assessed in a sub-sample of controls (without available bone marrow aspirates). Of the 757 controls, 43 children were randomly selected for hepcidin assessment. Hematological status of this sub-group was comparable to all controls (data not shown).

Written informed consent was obtained from a parent or guardian of each child. The study was approved by the Ethics Committees of the College of Medicine, Malawi, and the Liverpool School of Tropical Medicine, United Kingdom.

### Laboratory investigations

Hemoglobin, mean cellular volume (MCV) and mean corpuscular hemoglobin concentration (MCHC) were measured by a Coulter counter analyzer (Beckman Coulter, Durban, South Africa). Bone marrow slides were stained with Hematognost Fe (Merck, Darmstadt, Germany) and graded for intracellular iron content using a histological grading method which classifies iron status into six grades (0–6) [22]. In addition, all marrow smears were also assessed using an alternative grading method where utilizable iron was specifically assessed through determination of iron in the erythroblasts. At high power magnification (*1000) 20 fields surrounding the marrow fragments were examined and a hundred erythroblasts were examined to enumerate the percentage containing iron granules in their cytoplasm [23].

Within the first hour after collection, heparin plasma and serum aliquots were stored at −80°C. These samples were used for later (2009) assessment of hepcidin-25 (the bioactive, active form of the peptide). As outcome of assessments of plasma and serum hepcidin is comparable we will refer to concentrations as *serum* hepcidin. Hepcidin assessments were measured by a combination of weak cation exchange chromatography and time-of-flight mass spectrometry (WCX-TOF-MS) [24]. An internal standard (synthetic hepcidin-24; Peptide International Inc., Louisville, KY) was used for quantification [25]. Peptide spectra were generated on a Microflex LT matrix-enhanced laser desorption/ionization (MALDI) TOF-MS platform (Bruker Daltonics, Bremen, Germany). Serum hepcidin-25 concentrations are expressed as nanomoles per liter (nmol/L). The lower limit of detection of this method was 0.5 nmol/L [24]. Samples found to have a hepcidin concentration <0.5 nmol/L (57%) were imputed with a random value out of a uniform distribution with a minimum of 0.01 nmol/L and a maximum of 0.5 nmol/L. Ferritin was determined using electro-chemiluminescence immunoassay (Modular Analytics E170, Roche Diagnostics, Switzerland). Erythropoietin was determined using Immulite 2000 (maximum detection limit <200 and after dilution >1000 IU/L; Siemens DPC, New Jersey, USA). (Immunoturbidimetric assay (Modular P800, Roche Diagnostics, Switzerland) was used to determine serum transferrin and C-reactive protein (CRP). Soluble transferrin receptor (sTfR) was measured using an enzyme immunoassay (Ramco Laboratories, TX, USA, detection limit 1.0 µg/l). Interleukin-6 (IL-6) was measured by Cytometric Bead Array on a FACS- Calibur flow- cytometer (Becton-Dickson, South Africa). Clinical malaria was defined as a positive blood slide with concurrent fever (axillary temp >37.5°C), or history of fever within the previous 48 hours.

### Definitions

Deficiency of bone marrow iron stores was defined as grade 0 or 1 [22], [26]. Erythroblast iron incorporation was used as a proxy for utilizable iron in the bone marrow; insufficient erythroblast iron incorporation (functional bone marrow iron deficiency) was defined as less than 30% of the erythroblasts having visible iron granules while having replete iron stores [23]. Cut-off values for iron deficiency of the peripheral iron markers were as follows: hepcidin<0.5 nmol/L [27]; ferritin<30 µg/L [28]; sTfR>8.3 µg/ml; MCHC<32 g/L [3]; MCV<67 fl (<2 years old) and <73 fl (2–5 years old) [3]. Soluble transferrin log-ferritin index (sTfR-F index) was defined as [sTfR ÷ log ferritin] in which log refers to ‘base-10 log’ [29]. Using the cut-offs sTfR>8.3 µg/L and ferritin<30 µg/L, a cut-off for sTfR-F index was calculated (>5.6).

### Statistical Methods

Data were analyzed with use of SPSS 20.0 and AMOS 20.0 statistical computer packages (SPSS, Chicago, Illinois). Correlations with bone marrow iron gradings were assessed with the Kendall rank correlation test for ordinal data. Median hepcidin levels between the different sub-groups were compared using the Mann-Whitney U test. Using the dichotomous outcome of bone marrow iron status and erythroblast iron incorporation, receiver operating characteristics (ROC) curves and corresponding areas under the curve (AUC^ROC^) were created for hepcidin and conventional iron markers. The ROC curves were used to assess a cut-off for hepcidin providing optimal sensitivity and specificity in predicting bone marrow iron deficiency, and for all iron makers in predicting insufficient erythroblast iron incorporation.

To evaluate different signaling pathways of hepcidin, all relevant available covariates relating to hepcidin were analyzed using linear regression models with bone marrow iron deficiency as outcome. These predictors of hepcidin included erythropoietin and reticulocytosis (erythropoiesis), hemoglobin (hypoxia), bone marrow iron grading (iron status), CRP, IL-6, malaria and bacteremia (inflammation). Multivariate linear regression was performed with a model predicting log-hepcidin including one proxy variable for each pathway (bone marrow iron status, CRP, hemoglobin and erythropoietin).

To evaluate the association between hepcidin and erythroblast iron incorporation, all relevant available covariates relating to erythroblast iron incorporation (hepcidin, use of hematinics in the previous 4 weeks and CRP) were analyzed using univariate and multivariate logistic regression.

Missing observations were included in analyses by creating missing-value categories. Reported *p*-values are two-sided; statistical significance was set at the conventional 5% level.

#### Structural equation model

More complex multivariate analyses allowing interaction of covariates were performed using structural equation modeling [30]. This model was created containing all possible associations between all relevant available variables relating to hepcidin, plus malaria and bacteremia. Associations are indicated by standardized regression coefficients. All non-significant associations (p≥0.05) were removed. Relevant non-significant coefficients were retained and are displayed as dashed arrows. C-reactive protein was adjusted for malaria and bacteremia; interleukin 6 was adjusted for bacteremia. Skewed variables were log-transformed. Missing observations were considered as missing at random and were imputed by single imputation [30].

## Results

Of the 381 severely anemic children enrolled in the study, 237 underwent a bone marrow aspirate. In 139 of these children a serum sample with sufficient volume for hepcidin assessment was available. Hematological status of children with or without hepcidin and/or bone marrow assessment was comparable (data not shown). In Table 1 baseline characteristics of the severely anemic study group and the non-severely anemic control group are presented. Bone marrow iron deficiency, inflammation (CRP>40.0 mg/L) and high levels of erythropoietin (epo>1000 u/L), were found in 30.2%, 81.2% and 95.6% of severely anemic children, respectively. The median value of hepcidin in the severely anemic children was 0.40 nmol/L (IQR 0.19–2.10 nmol/L), and 1.40 nmol/L (0.31–5.60 nmol/L) in the non-severely anemic control group (*p = 0.04*).

**Table 1:** Baseline characteristics of the study population.

Characteristic		severely anemic cases	non-severely anemic controls
**Demographics**	N	207	43
	Male	48.8% (101/207)	69.8% (30/43)
	Age (months)	14.0 (9.7–26.3)	20.1 (10.6–28.3)
**Hematological status**	Hemoglobin (g/dl)	3.6 (0.8)	9.7 (1.9)
	MCHC (g/dl)	32.60(6.8)	33.2 (5.4)
	MCV (fl)	82.7 (15.4)	71.2 (8.0)
	Reticulocytes %	3.6 (2.1–6.7)	2.3 (1.7–4.3)
	Erythropoietin >1000 U/L	95.6% (108/113)	0% (0/39)
**Iron status**	Deficiency of bone marrow iron stores	30.2% (42/139)	n/a
	Insufficient erythroblast iron incorporation	41.4% (48/116)	n/a
	Hepcidin (nmol/L)	0.40 (0.19–2.10)	1.40 (0.31–5.60)
	Ferritin (µg/l)	404 (157–905)	50 (12–171)
	sTfR (µg/ml)	13.4 (8.1–22.4)	12.5 (7.2–17.5)
	sTfR-F index	5.1 (3.1–9.6)	5.9 (3.1–12.0)
**Inflammatory status**	Bacteremia	12.6% (25/198)	0% (0/20)
	Malaria	59.9% (124/207)	37.2% (16/43)
	HIV infected	7.7% (15/195)	3.7% (2/43)
	CRP>40 mg/L	81.2% (159/196)	37.7% (12/31)
	Interleukin-6 pg/ml	41.6 (20.9–142.3)	25.6 (10.0–67.3

Deficiency of bone marrow iron stores was defined as a bone marrow iron score of 0 or 1; Deficiency of functional bone marrow iron was defined as <30% iron deficient erythroblasts while having replete iron stores [22], [26]; CRP: C-Reactive Protein; HIV: Human Immunodeficiency Virus; MCHC: mean cell hemoglobin concentration; MCV mean corpuscular volume; sTfR: soluble transferrin receptor; sTfR-F index: sTfR-log ferritin index^31^Normally distributed variables are presented with their mean value (s.d.); skewed variable are presented with their median value (inter quartile range). Malaria was defined as a positive blood slide with concurrent fever (axillary temp >37.5°C), or history of fever within the previous 48 hours.

### Hepcidin predicting bone marrow iron status and erythroblast iron incorporation

Hepcidin levels poorly correlated with bone marrow iron grading (Kendall rank correlation coefficient 0.05, *p = 0.5*). The ROC-curve for hepcidin identifying bone marrow iron deficiency showed an area under curve (AUC^ROC^) of 0.598, marginally higher than 0.500 (*p = 0.07*), the cut-off to indicate an effective test. The optimal cut-off for hepcidin to predict bone marrow iron deficiency, derived from the ROC-curve, was 0.5 nmol/L. Using this cut-off, hepcidin sensitivity and specificity estimates to detect bone marrow iron deficiency were 66.7% and 49.5% respectively. The AUC^ROC^, sensitivity and specificity of ferritin, sTfR, sTfR-F index and MCV and MCHC were additionally assessed and are shown in table 2.

**Table 2:** Performance of biochemical iron markers to identify children with deficiency of bone marrow iron stores or insufficient erythroblast iron incorporation.

	*Bone marrow iron deficiency*								*Insufficient erythroblast iron incorporation*							
Iron marker	AUCROC	*p value*	Sensitivity (%)	95% CI		Specificity (%)	95% CI		AUCROC	*p value*	Sensitivity (%)	95% CI		Specificity (%)	95% CI	
Hepcidin (nmol/L)	0.598	0.07	66.7	50,5	80,4	49.5	39,2	59,8	0.591	0.1	54.2	39,2	68,6	61.8	49,2	73,3
Ferritin (ug/l)	0.855	<00001	41.7	22,2	63,3	98.1	87,7	99,7	0.524	0.8	48.0	28,7	68,0	54.1	36,9	70,5
sTfR (ug/ml)	0.823	<00001	92.5	79,6	98,4	35.5	25,8	46,1	0.634	0.02	58.7	43,2	73,0	57.6	44,8	69,7
sTfR-F index	0.815	<00001	75.0	53,3	90,2	73.5	58,9	85,0	0.577	0.3	96.2	80,3	99,4	13.9	4,7	29,5
MCHC (g/dl)	0.727	<00001	61.5	46,5	80,2	68.4	57,9	78,9	0.447	0.4	45.0	29,3	61,5	50.9	37,1	64,6
MCV (fl)	0.489	0.9	22.9	10,5	40,1	90.1	81,5	95,6	0.676	0.003	70.0	53,5	83,4	58.9	45,0	71,9

Deficiency of bone marrow iron stores was defined as a bone marrow iron score of 0 or 1; CRP: C-Reactive Protein; HIV: Human Immunodeficiency Virus; MCHC: mean cell hemoglobin concentration; MCV mean corpuscular volume; sTfR: soluble transferrin receptor; sTfR-F index: sTfR-log ferritin index. Insufficient erythroblast iron incorporation: defined as <30% iron deficient erythroblasts. AUC area under the curve; ROC: receiver operating characteristics.

**Figure 1:**
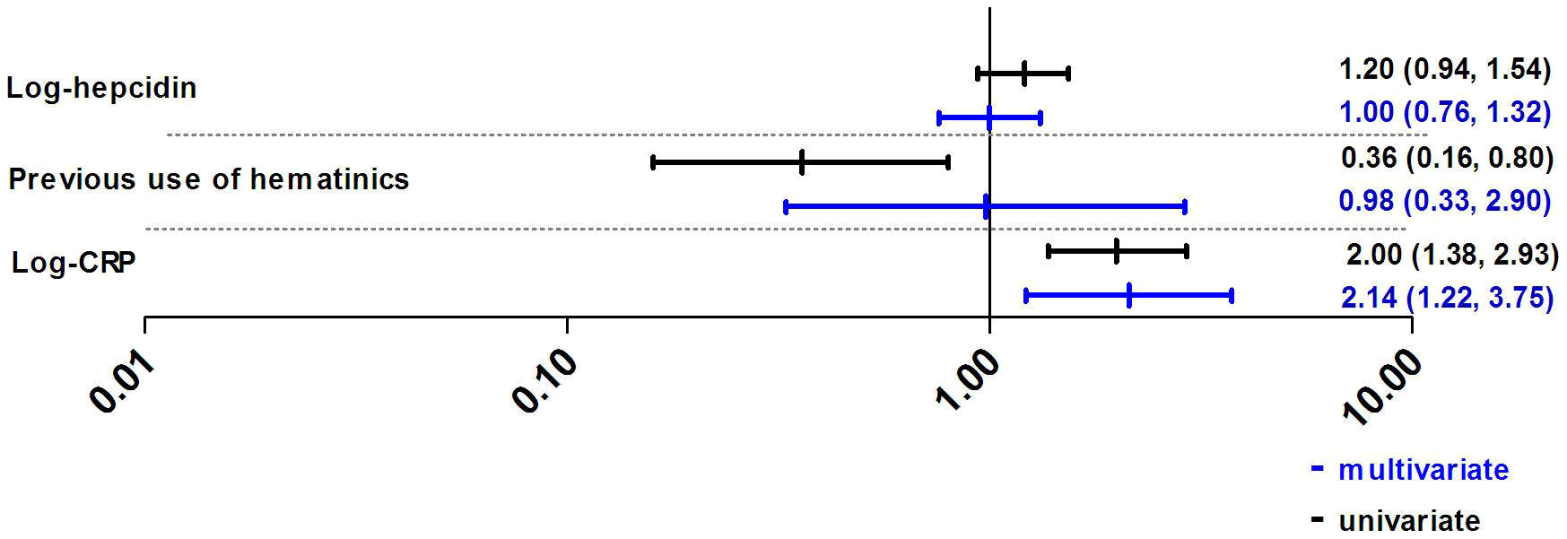
Univariate and multivariate logistic regression analyses with all relevant covariates predicting erythroblast iron incorporation. Regression coefficients are presented with their 95% confidence intervals. Insufficient erythroblast iron incorporation was defined as less than 30% erythroblasts having visible iron granules while having replete iron stores [23]. CRP: c-reactive protein. Previous use of hematinics is defined as use of iron supplements in the previous four weeks.

**Figure 2:**
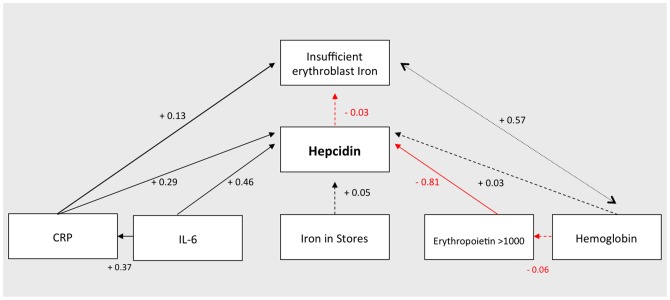
Univariate and multivariate linear regression analyses with all relevant covariates predicting log hepcidin. Regression coefficients are presented with 95% confidence interval. Bone marrow iron stores was scores in a range from 0 to 6 [22]. CRP: c-reactive protein; IL-6: interleukin 6. Bacteremia was analyzed as dichotomous variable. Malaria was defined as a positive blood slide with concurrent fever (axillary temp >37.5°C), or history of fever within the previous 48 hours.

Of the 139 available bone marrow smears, 23 were excluded from the erythroblast iron incorporation analyses because of poor staining. Of the remaining samples, 49 (41.4%) showed insufficient erythroblast iron incorporation. The assessments of hepcidin and conventional iron markers to predict erythroblast iron incorporation are shown in table 2. The AUC^ROC^ for hepcidin was 0.591 (*p = 0.1*). Using the optimal cut-off of 0.5 nmol/L, sensitivity and specificity of hepcidin to predict insufficient erythroblast iron incorporation were 54.2% and 61.8%, respectively (Table 2). The univariate and multivariate logistic regression analyses to model the relation between hepcidin and erythroblast iron incorporation are shown in figure 1. Applying these variables in the structural equation model (figure 2) it was found that CRP, but not hepcidin levels, were positively associated with insufficient erythroblast iron incorporation (regression coefficient of 0.13, *p = 0.02*, and −0.03, *p = 0.6* for CRP and hepcidin, respectively). The overall root mean square area of approximation, an indicator for model fit, was 0.288 (95% CI 0.272–0.304). Exclusion of non-significant associations gave a virtually identical model'

### The prioritizing of the four hepcidin signaling pathways

**Figure 3:**
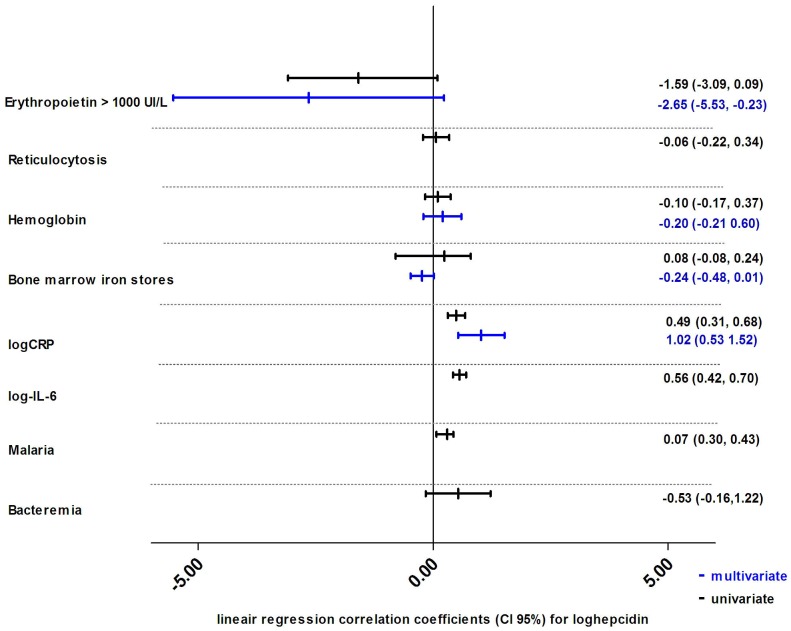
Structural equation model for hepcidin, hypoxia/erythropoiesis, iron status and inflammation. In this exploratory model of the factors associated with serum hepcidin, the sizes of the associations are indicated by the standardized regression coefficients. An inverse association is indicated by a red line. This model was created containing all possible associations between the displayed variables, after which all non-significant arrows (*p*≥0.05) were removed. A few non-significant correlations, deemed relevant by the authors, were retained in the figure, displayed as dashed arrows. C-reactive protein (CRP) was adjusted for malaria and bacteremia; interleukin 6 (IL-6) was adjusted for bacteremia (omitted for clarity). Iron in stores was defined as bone marrow iron score (0–6) [22]. Insufficient erythrocyte iron incorporation was defined as less than 30% erythroblasts having visible iron granules while having replete iron stores. Erythroblast iron was defined as more than 30% of the erythroblasts having visible iron granules while having replete bone marrow iron stores [23]. The overall root mean square area of approximation, an indicator for model fit, was 0.288 (95% CI 0.272–0.304).

Univariate and multivariate associations of the available variables belonging to the four signaling pathways [12] of hepcidin are illustrated in figure 3. The *iron pathway*, represented by bone marrow iron stores had a weak association with hepcidin (regression coefficient 0.05, *p = 0.6*). The *inflammation pathway*, represented by CRP and IL-6, was strongly positively associated with hepcidin (regression coefficients of 0.29, *p<0.0001* and 0.46, *p<0.0001* respectively). CRP was adjusted for malaria and bacteremia; IL-6 was adjusted for bacteremia. The *hypoxia pathway* reflected by hemoglobin concentration was not associated with hepcidin (regression coefficient +0.03, *p = 0.7*). For the *erythropoietic pathway*, erythropoietin had a strong negative association with hepcidin (regression coefficient −0.81, *p<0.0001*). The overall root mean square area of approximation, an indicator for model fit, was 0.288 (95% CI 0.272–0.304). Exclusion of non-significant associations gave a virtually identical model'

## Discussion

This is the first study evaluating hepcidin and bone marrow iron status in severely anemic African children. Hepcidin levels were largely undetectable, probably as a result of a dominance of the erythropoietin signal for hepcidin. As a consequence, hepcidin poorly correlated with the iron pathway and was therefore not a good marker for iron deficiency. Furthermore, it was found that hepcidin was also not associated with the incorporation of iron into the erythroblast.

Previous studies investigating the relation between iron status and hepcidin showed a positive association between hepcidin and serum ferritin and liver iron levels [14], [15], [31]. With the high prevalence of iron deficiency in these children we would have expected low hepcidin levels. Although levels were low, the structural equation model clearly showed that hepcidin was only weakly associated with bone marrow iron stores. This may be explained by the influence of the other signaling pathways on hepcidin production, as erythropoietic and inflammatory pathways were strongly associated with hepcidin. In view of these findings hepcidin was not the preferred marker for iron status assessment. The soluble transferin receptor-log ferritin index (TfR-F index) was the more suitable [21], although still not ideal.

Erythropoietin levels were high in these severely anemic children, and strongly associated with low hepcidin levels. The regression coefficients determined in the structural equation model, and occurrence of low hepcidin levels despite a high incidence of infection, suggested that the down-regulating effect of erythropoietin exceeded the up-regulating effect of inflammation. This interpretation is supported by the observation of higher hepcidin with lower erythropoietin values, and higher CRP and IL-6 levels in the control group. Erythropoietin dominating inflammation has also been reported in an infectious murine model in which low hepcidin values were observed following administration of erythropoietin [32]. Similar findings were reported in humans although these subjects had less inflammation compared to children in the present study [33], [34]. It is unclear whether erythropoietin is directly down regulating hepatic hepcidin synthesis, or indirectly through induced erythropoiesis and the previously proposed associated erythropoietic factors, i.e. growth differentiation factor-15 (GDF15) and twisted gastrulation protein homolog-1 (TWSG1) [35].

Hypoxia may directly down regulate hepcidin release [36]. It was therefore expected that hemoglobin and hepcidin concentrations would be positively correlated which was not observed. An explanation could be that the severity of anemia may have reduced the heterogeneity of the hepcidin response. As a regulator of available (utilizable) iron, hepcidin was also expected to be positively associated with erythroblast iron incorporation. This association was not observed, which is in contrast with findings in Gambian children after 14 days of oral iron supplementation [17]. Our findings are consistent with those of Cercamondi *et al*, who reported that hepcidin was associated with intestinal iron absorption, but not with red blood cell iron incorporation [37]. These differences may relate to intermediate factors interfering with iron incorporation and explain the lack of correlation between hepcidin and erythroblast iron incorporation in the present study. Furthermore, iron availability (through absorption and release from storage), and iron incorporation are dynamic processes which may be difficult to profile using cross-sectional data. Inflammatory factors, other than hepcidin, may inhibit erythroblast iron incorporation as this was not correlated with hepcidin, malaria, or IL-6, but was significantly associated with raised CRP values.

### Considerations and limitations

The majority (57%) of severely anemic children in this study had hepcidin values <0.5 nM, which might indicate hepcidin production was completely “switched off”. Analyses were therefore repeated with hepcidin as a dichotomous value which showed comparable outcomes. A more sensitive test with a lower hepcidin detection limit may provide an improved correlation between hepcidin and iron status, but this is a general limitation of current hepcidin assays.

Ideally the fit of the structural equation model, represented by the root mean square area of approximation (RMSEA), would be smaller then 0.050. Our RMSEA was 0.288 which may be explained in part by missing values. Application of this model to a similar but complete data set should decrease the RMSEA and provide a stronger hypothetical model. Another explanation for the high RMSEA approximation could be a missing intermediate model variable, such as an unmeasured erythropoietic or inflammatory factor.

It is important to note that variation in bone marrow findings can result from an uneven distribution of iron in the bone marrow [20]. Therefore a minimum of 10 bone marrow particles per aspirate was examined to optimize sensitivity of the bone marrow examination [38]. Bone marrow assessment is still generally considered the most reliable diagnostic test for iron status [20].

### Clinical implications

**Figure 4:**
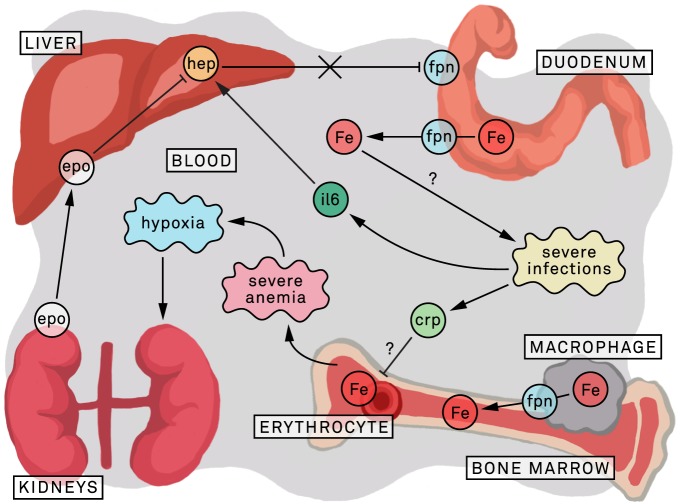
Hypothetic model of the iron metabolism in severely anemic children. Severe anemia is associated with severe infections [7]; the associated high levels of IL-6 induce hepcidin [12]. Although in severe anemia hypoxia stimulates the kidneys to produce erythropoietin; the erythropoietin induced erythropoietic drive will down-regulate hepatic hepcidin expression, and may overrule hepcidin stimulation of IL-6. The resulting low hepcidin values are associated with decreased degradation of the cellular iron exporter ferroportin; in iron replete subjects or during iron supplementation/treatment this will lead to a rise in plasma iron levels [12]. In infection endemic areas this may increase infection risk [5], [40]. In addition inflammatory related factors possibly inhibit erythroblast iron incorporation. CRP: c-reactive protein; ery: erythroblast; epo: erythropoietin; Fe: plasma iron; FPN: ferroportin; hep: hepcidin-25; IL-6; interleukin 6.

These findings raise concerns with respect to iron supplementation and treatment in severely anemic children living in malaria endemic areas with high infection pressure. In these severely anemic children hepcidin concentrations were low, despite evidence of inflammation in the laboratory findings. With inflammation a rise in hepcidin would be expected, which would diminish iron absorption and availability for pathogens, including malaria parasites [39]. Iron supplementation/treatment , as currently recommended for severely anemic children [6], could in enhance infection risk in these children (figure 4). There is a need for studies investigating iron uptake using stable isotopes in severely anemic children with malaria, or other infectious diseases, and to assess infection incidence in children with low hepcidin levels receiving iron supplementation.
